# Effect of the Addition of Freeze-Dried Grape Pomace on Fresh Tagliatelle Gluten Network and Relationship to Sensory and Chemical Quality

**DOI:** 10.3390/foods12142699

**Published:** 2023-07-13

**Authors:** Barbara la Gatta, Mariacinzia Rutigliano, Maria Teresa Liberatore, Flavia Dilucia, Maurizio Palmitessa, Aldo Di Luccia, Carmela Lamacchia

**Affiliations:** 1 Department of Sciences of Agriculture, Food, Natural Resources and Engineering (DAFNE), University of Foggia, Via Napoli 25, 71122 Foggia, Italy; barbara.lagatta@unifg.it (B.l.G.); mariacinzia.rutigliano@unifg.it (M.R.); mariateresa.liberatore@unifg.it (M.T.L.); flavia.dilucia@unifg.it (F.D.); aldo.diluccia@unifg.it (A.D.L.); 2 NEWTRA FOOD s.r.l. Company, Via degli Artigiani 67, 76121 Barletta, Italy; info@newtrafood.it

**Keywords:** grape pomace, pasta, gluten structure, antioxidant activity, polyphenol content, sensory properties

## Abstract

The incorporation of 5 and 10% freeze-dried grape pomace powder (GPP) in fresh tagliatelle pasta preparation was evaluated for its effect on chemical composition, gluten protein structure, and sensory properties. The addition of the freeze-dried GPP led to a significant increase (*p* < 0.05) in polyphenol content in the raw and cooked fortified pasta samples with respect to 100% semolina pasta, although the phenolic content decreased after the cooking process. The opposite phenomenon was observed with the antioxidant activity, which increased significantly (*p* < 0.05) when switching from raw to cooked pasta samples fortified with GPP. The formation of a proper gluten structure was found in the fortified raw pasta, even if a change in the protein arrangement was shown in the fortified cooked samples, confirmed by a significant reduction (*p* < 0.05) in both the unextractable polymeric protein percentage (% UPP) and disulfide bond (S-S) formation. These results suggest a possible interaction between the protein sulfhydryl groups (-Cys) and polyphenols of grape pomace during cooking through non-disulfide covalent bonds, which was confirmed by the significant (*p* < 0.05) decrease in the -SH groups when comparing 100% semolina pasta with fortified pasta sample. Finally, a sensory analysis showed that the highest significant score (*p* < 0.05) was achieved by the 5% GP-fresh pasta sample.

## 1. Introduction

Food is no longer only intended to satisfy hunger and provide necessary nutrients, but it is also used to prevent nutrition-related diseases and improve physical and mental well-being [1]. The food industry has faced technical and economic changes both in society and food processing, which, in turn, have had a significant impact on the entire supply chain, from food distribution to the end consumers. Moreover, these changes have forced companies to pay considerable attention to food products that meet the consumer demands for a healthy lifestyle. In recent years, consumer demand for nutritional diets rich in compounds with functional properties has increased, as several pieces of research have shown the beneficial effects of those properties for human health, as well as preventing and fighting several diseases [2].

Grapes have worldwide economic and nutritional importance. They are phenolic-rich fruits, presenting a protective effect against low-density lipoprotein (LDL) oxidation, an improvement in coronary blood flow, and other beneficial effects for human health [3]. Approximately 75% of produced grapes is intended for wine production, out of which 20–30% represents a waste product [4]. This waste is also called grape pomace, which is a residue from grape processing during wine production, consisting of the skins, remaining pulp, seeds, and stalks [5]. Grape pomace contains significant amounts of substances that can be considered beneficial to health [6], such as phenolic compounds (anthocyanins, flavan-3-ols, stilbenes, and phenolic acids) [7] and dietary fiber, mostly depending on the grape variety [8]. Owing to the high-water content of this product, it needs to be frozen until use and/or dried [9] before application for the formulation of high-value food products.

Recently, many pieces of research have been carried out by testing the fortification of pasta products with food by-products [10] in order to exploit the enhanced nutritional properties of these ingredients. Pasta lends itself well to modification according to various recipes, as it is accepted worldwide due to its low cost, ease of production, and sensory attributes. From a technological point of view, the exclusive use of durum wheat semolina ensures some desirable parameters in cooked pasta, such as good texture, resistance to surface disintegration, and retention of a firm structure [11]. Thus, fortification can affect the technological properties of pasta [10], mainly because the addition of new ingredients can alter gluten network formation and, consequently, pasta structure, with an effect on sensory analysis.

From a commercial point of view and despite the growing interest in the healthy features of food products, good sensory properties still remain a key priority as a consumer choice criterion.

In this context, the addition of grape pomace for the formulation of durum wheat pasta has already been studied by Tolve et al. [11], who highlighted an increase in the total polyphenol content and antioxidant activity of fortified pasta samples, showing the high potential of this by-product.

To the best of our knowledge, no studies about the fortification of fresh pasta with grape pomace regarding the quality of the gluten network and its relationship with sensory properties and antioxidant activity have been found in the literature. Fresh pasta is a traditional homemade product, which can be easily prepared, but the absence of the drying step [12] represents an important factor to take into account in order to evaluate how pasta performance and chemical characteristics can change when another ingredient is added to the formulation.

In light of these considerations, the aim of this work was an evaluation of the effect of fortification (5% and 10%) with grape pomace powder (GPP) on the gluten network arrangement of fresh tagliatelle pasta samples and the relationship with its chemical and sensory properties.

The use of a novel, patented, green process applied to the wet grape pomace (GP) was tested in order to exploit the nutritional features of this by-product, used as an ingredient. Therefore, the total polyphenol content, antioxidant activity, and polymeric protein distribution and extractability (Unextractable Polymeric Proteins (% UPP) were studied using size-exclusion high-performance liquid chromatography (SE-HPLC), and the disulfide bond content of newly formulated fresh pasta samples was studied. Finally, the sensory properties of fortified fresh pasta samples were evaluated.

## 2. Materials and Methods

### 2.1. Grape Pomace Powder Preparation

Grape pomace (var. Montepulciano) was supplied by a local winery (Antica Cantina, San Severo, Foggia, Puglia), in production year 2022, and they were stored at −20 °C to avoid the enzymatic degradation of the polyphenols until use. Samples (free from impurities) were divided into 3 groups:

1. Fresh sample, used as it was;
2. Dried sample, obtained following a drying process carried out at 70 °C for 16 h in a tray dryer, and used as a powder;
3. Freeze-dried sample, obtained by following the application of a patented, non-thermal, and non-invasive procedure and subsequently freeze-dried (Patent No. 001426984), and used as powder.

### 2.2. Pasta Production

**Figure 1:**
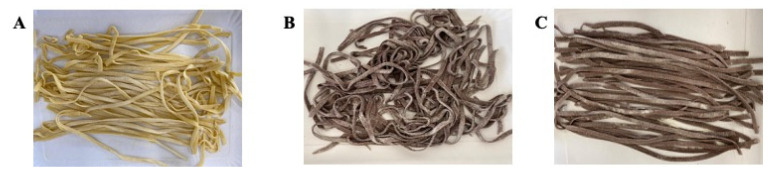
Fresh pasta samples. (**A**) 100% Durum wheat semolina fresh pasta; (**B**) 5% Grape pomace powder fresh pasta; (**C**) 10% Grape pomace powder fresh pasta.

Durum wheat semolina (SEM) (Granoro, Italy, production 2021) was purchased at local markets. Durum wheat semolina (SEM) contained 71.0% of total available carbohydrates, 12.5% crude proteins, 1.5% total fat, and 3.0% total dietary fiber, as reported on the label. Fresh pasta samples, such as tagliatelle, were hand-made and the doughs were prepared by mixing durum wheat semolina with water (30%), to obtain the control sample (100% semolina fresh pasta) and the fortified samples, adding 5% and 10% of the freeze-dried grape pomace powder (GP-fresh pasta) obtained as previously described, respectively. Fresh pasta samples were then put on a vessel, covered with a cotton cloth, and left to dry at room temperature until it was reached a moisture content in a range between 26 and 28%, according to Italian legal requirements for fresh pasta [13]. Fresh pasta samples are shown in Figure 1.

### 2.3. Chemical Analysis

#### 2.3.1. Methanolic Extraction

The extraction of phenolic compounds from grape pomace powders and pasta samples was performed according to Farhadi et al. [14] with minor modifications. One gram (1 g) of powder and crushed samples were mixed with 5 mL methanol/HCl 99:1 (*v*/*v*) and placed into 50 mL vials, which were immersed in an ultrasound bath and subjected to ultrasound treatment at room temperature for 20 min. After that, samples were left at room temperature for about 15 min and then were centrifuged at 7500× *g* for 10 min. The above-mentioned method was carried out in triplicate and all the supernatants were collected in a 20 mL flask and then stored at −20 °C until the analyses.

#### 2.3.2. Determination of Total Polyphenols Content (TPC)

The total polyphenols content (TPC) of the methanolic extracts was measured by the Folin–Ciocalteu method according to the procedure described by Farhadi et al. [14]. Gallic acid was used to calculate the standard curve (5–500 ppm). The results were expressed as mg of gallic acid equivalents (GAE) per 100 g of pasta. The determination was performed in triplicate.

#### 2.3.3. Polyphenolic Profile by High-Performance Liquid Chromatography (HPLC) 

A liquid chromatograph Agilent 1200 Series system (Santa Clara, CA, USA) equipped with a Multospher 100 P 18 column (5 μm, 125 × 4.6 mm, Chromatographe-Service GmbH, Langerwehe, Germany) set at 20 °C was used to separate and quantify the polyphenols of the grape pomace powders, as raw, dried, and freeze-dried samples. Before the analysis, 1 mL of methanolic extract was dried and resuspended in 1 mL of mobile phase (A) and an aliquot (100 μL) was injected into the column. The analysis was performed according to Revilla et al. [15], with minor modifications. Separations were carried out using a flow rate of 0.5 mL/min and the detection was set at 280 nm and 313 nm. The gradient separation was achieved using the following mobile phases: (A) 95:5 (*v*/*v*) ultra-pure water: acetonitrile (*v*/*v*) and (B) 50:50 (*v*/*v*) ultra-pure water: acetonitrile solution, brought to pH 1.8 with hydrochloric acid. The analysis was programmed to go from 5% to 100% of mobile phase (B) in 48 min and maintain this condition for 10 min, after that the initial condition was restored. The chromatographic run lasted 70 min.

#### 2.3.4. DPPH Radical Scavenging Activity 

The free-radical scavenging activity of the methanolic extracts was analyzed by using the 2,2-diphenyl-1-picryl-hydrazyl (DPPH) method, according to the procedure described by Farhadi et al. [14].

The determination was performed in triplicate for all the samples. Trolox was used to calculate the standard curve (5–500 ppm) and the results were expressed as μmol of TEAC (Trolox Equivalent Antioxidant Capacity) per 100 g of pasta.

### 2.4. Protein Content

The protein content of milled tagliatelle was obtained by the Kjeldhal method by using a conversion factor of 6.25. The analyses were carried out by an automatic digestion unit (60 min at 420 °C) and through an automatic distillation and titration system (UDK159, VELP Scientifica Srl, Usmate, Monza-Brianza, Italy). 

### 2.5. Size-Exclusion Liquid Chromatography Analysis (SE-HPLC)

Polymeric proteins from semolina, freeze-dried GPP, and fresh pasta samples were fractionated through size-exclusion high-performance liquid chromatography (SE-HPLC) using a Phenomenex Biosep TM SEC 4000 column (Phenomenex) [16]. The analysis was carried out with a two-step extraction procedure [17]. The first step extracted the SDS-extractable proteins (soluble in diluted SDS), whilst the second extract contains the SDS-unextractable proteins (proteins soluble only after sonication). The extracted proteins were separated on SE-HPLC by loading 20 μl of sample into an eluant of 50% (*v*/*v*) acetonitrile and ultra-pure water containing 1% (*v*/*v*) trifluoracetic acid (TFA) at a flow rate of 0.7 mL/min for 30 min. Proteins were detected at a wavelength of 214 nm. Three replicates of each sample were used for the investigation of protein composition. The percentage of unextractable polymeric protein (%UPP) was calculated as described by Gupta et al. [17]. Briefly, the percentage of total UPP was calculated as [peak 1 + 2 area (unextractable)/peak 1 + 2 area (total)] × 100. Peak 1 + 2 (total) refers to the total of peak 1 + 2 (extractable) and peak 1 + 2 (unextractable) [16]. The SE-HPLC column was calibrated using protein standards with a range of molecular weights (KDa) as follows: ribonuclease A (13.7), chymotripsinogen (25.0), ovalbumin (43.0), bovine serum albumin (67.0), aldolase (158), catalase (232), ferritin (440), and thyroglobulin (669). 

### 2.6. SDS–PAGE (Sodium Dodecyl Sulphate–Polyacrylamide Gel Electrophoresis) Analyses

The total proteins from durum wheat semolina and fresh pasta samples were extracted (1:10, (*w*/*v*)) with a buffer containing Tris-HCl 0.0625 M pH 6.8, SDS 2% (*w*/*v*), Glycerol 10% (*v*/*v*). Samples were left in contact with the extraction buffer for 3 h and then they were centrifuged at 13,000× *g* for 10 min at 25 °C. The supernatants were carefully removed and stored at −20 °C until the analysis. SDS-PAGE was performed both under reducing conditions with 5% (*w*/*v*) dithiotreitol (DTT) and non-reducing conditions, on a 11% polyacrylamide gels (8.6 × 6.7 cm) using a Mini-PROTEAN Tetra system electrophoresis cell (Bio-Rad, Hercules, CA, USA). The electrophoretic analyses were performed using a running buffer consisting of: Tris 0.025 M, Glycine 20 mM, 1% SDS. Electrophoresis was carried out by applying a potential of 200 V and lasted one hour, using a Prestained standard SDS-PAGE molecular weight standard (Bio-Rad, Richmond, CA, USA). The gels were stained with a 0.25% (*w*/*v*) solution of Coomassie Brilliant Blue G-250 (CBB) and fixed with a 7% (*v*/*v*) solution of acetic acid and 40% (*v*/*v*) of methanol and destained with water.

### 2.7. Determination of SH and S–S Groups

Free thiols and disulphide groups in the semolina flour and pasta samples were assessed by a colorimetric determination, using a solid phase assay NTSB^2−^, according to the method described by Chan and Wassermann [18]. The determinations were performed in triplicate.

### 2.8. Pasta Cooking Test

The pasta was cooked in boiling water, without the addition of salt. The optimal cooking time (OCT) was evaluated by following the method by Padalino et al. [19]. The determination was performed in triplicate for all samples. 

### 2.9. Sensory Analysis

In order to evaluate the acceptability of the new formulations of fresh pasta for consumers, a sensory analysis was carried out by a panel of 20 judges (*n* = 20). Before the sensory assessment, all participants evaluated different attributes: odor, color, superficial aspect, general presentation and overall acceptability. Furthermore, cooked pasta samples were evaluated for cooking water, surface stickiness, chewiness, and elasticity. Sensory attributes were evaluated using a five-point (1 to 5) hedonic scale ranging from extremely unpleasant (1) to extremely pleasant (5).

### 2.10. Statistical Analysis

One-way ANOVA test was used to evaluate the effects of the addition of freeze-dried grape pomace powder on the chemical and technological characteristics of pasta samples and the difference were evaluated by the Tukey test. XLSTAT 2020.1 software (Addinsoft, New York, NY, USA) was used to perform the tests. 

## 3. Results and Discussion

### 3.1. Chemical Characteristics of Grape Pomace Powders and GP-Fresh Pasta

In order to have a better understanding of the potential of the grape pomace powders, the total phenolic (mg gallic acid/100 g dw) content and antioxidant activity, measured by DPPH assay of raw, dried, and freeze-dried GPP were shown in Table 1a. 

The results indicated that the GPP extracts had a high content of polyphenols, 3191, 7380 and 18,199 mg gallic acid/100 g dw, respectively. These values were greater than those reported by Gonz’alez-Centeno et al. [20], who studied the effect of acoustic frequency, ultrasonic power density, and extraction time of grape pomace by aqueous ultrasonic-assisted extraction (UAE), but also greater than those reported by Marinelli et al. [21], who studied the fortification of spaghetti with aqueous grape marc extract. Moreover, the extracts showed high antioxidant activity, revealing 1417, 1303, and 1523 *μ*mol TEAC/100 g dw, respectively, although the differences were not significant (Table 1a). The highest value was registered for the freeze-dried GPP, which showed a significant (*p* < 0.05) higher content of total polyphenols than the dried one, and as expected, the raw matter. These results could be due to the application of the new technology, already used by Dilucia et al. [22], for the preparation of vegetables to be used as powder for infusions preparation, which showed higher values for TPC and antioxidant activity, when the non-thermal and non-invasive procedure and subsequently freeze-drying process were applied. The use of the freeze-drying technique to dry grape pomace allows polyphenols and antioxidant compounds not to be deteriorated and inactivated by high temperatures, as happens with the application of drying technique [23]. The grape seeds dried at 100 and 140 °C, and showed a reduction of 18.6% and 32.6% of the extractable total polyphenols, respectively, as well as a reduced antioxidant activity of grape seeds when compared to freeze-dried results [24]. Moreover, the polyphenolic profile of the raw, dried, and freeze-dried grape pomace powders was determined by HPLC-DAD analyses (Figure S1), at 280 nm and 313 nm, because different classes of polyphenols could be found in this kind of matrix [7]. As expected, the chromatographic profiles showed that freeze-dried GPP (Figure S1, Panel E, F) showed, at both tested wavelengths, a higher number of peaks, with higher absorbances than raw GPP (Figure S1, Panel A, B) and dried GPP (Figure S1, Panel C, D), respectively. Further analyses are ongoing in order to better know the composition of the polyphenols of the freeze-dried-GPP powder. Based on the obtained results, we chose to use the freeze-dried GPP as a functional ingredient to be added to semolina flour (at 5% and 10%) and to prepare the fortified tagliatelle samples. 

Concerning the new-formulated fresh pasta samples (GP-fresh pasta), Table 1b showed that the phenolic content significantly increased (*p* < 0.05) with the increase in GPP percentage in the raw pasta, in agreement with the findings of Gaita et al. [25] and Sant’Anna et al. [26], who revealed an increase in the phenolic content of fortified fettuccini samples with different doses of grape pomace powder. 

The phenolic content of pasta samples significantly decreased (*p* < 0.05) with the cooking process compared to raw samples, except for the 100% semolina fresh pasta sample, which did not show a decrease. The same trend was observed by Marinelli et al. [20], despite the negative effect of the temperature on the polyphenolic content in our study that was limited by preparing pasta samples without the drying step. However, according to Abdel-Aal and Rabalski [27] the effect of cooking on phenols could be not always the same, depending on the type of bioactive compound and product.

According to the obtained data, the fortification of fresh pasta with freeze-dried GPP led to a significant increase (*p* < 0.05) in antioxidant activity compared to the 100% semolina fresh pasta sample, but the increase was not proportional to the percentage of GPP added and to the polyphenolic content (Table 1b). These results were in agreement with the data reported by Gaita et al. [25] and Marinelli et al. [21], who observed an increase in the antioxidant activity of spaghetti samples fortified with grape marc extract. It was noteworthy that the antioxidant activity of GP-fresh pasta sample shown in Table 1b increased (*p* < 0.05) with the cooking process with respect to the raw pasta samples, although, also in this case, the increase was not proportional to the increase in the percentage of GPP added, suggesting a saturation effect of the antioxidant activity. This unexpected result could be explained by taking into account the possible interaction between polyphenols and proteins or carbohydrates, since different studies showed that proteins or carbohydrates and polyphenols can bind each other with covalent bonds [28]. Moreover, Palermo et al. [29] reported that the interactions with the food matrix can preserve anthocyanins from cooking. This protection, which was not detectable through the assay of total polyphenols, could find its confirmation with the increase in the antioxidant activity highlighted in this study. Furthermore, it could be assessed that the application of the novel patented procedure before the freeze-drying process promoted the content of polyphenols and proteins, and the lower residual water content favored their interaction. This interaction represented a natural protection for the bioavailability of the involved biomolecules, allowing us to preserve their activity [28].

### 3.2. Effect of the Addition of Freeze-Dried GPP on Pasta Gluten Network

#### 3.2.1. Protein Content of Semolina- and GP-Fresh Pasta

The protein content of semolina, freeze-dried GPP and raw and cooked fresh pasta samples were shown in Table 2. In total, 100% Raw semolina fresh pasta exhibited roughly the same amount of protein as semolina flour (11.6%), while the cooked one revealed a 48% of protein loss during cooking (5.92%), probably due to a faster starch swelling that slowed down the rate of protein interaction, leading to a weakening of the protein network [30]. It is well established that the drying step appears to be essential to keep the pasta structure [30,31], especially during overcooking; the protein network of a low temperature (55 °C) dried pasta is partially disrupted and loses its continuity after the cooking procedures.

On the contrary, the application of a very high-temperature (100 °C) process and a low moisture level allows us to preserve the microstructure of pasta, even after a prolonged cooking phase [32]. In our study, the lack, or at least the lower protein interaction, was probably accentuated by the absence of the drying process. Raw GP-fresh pasta sample, both with the addition of 5% and 10% GPP, showed a protein content similar to the 100% semolina fresh pasta sample (11.3% and 10.9%, respectively), being the grape pomace mainly characterized by the presence of grape seed proteins that can range from 6% to 15% [23].

The major protein component in grape seed protein isolate was found to be a globulin-link protein with subunit molecular weights varying from 25.5 to 40.0 kDa, as determined by SDS-PAGE [33]. Cooked GP-fresh pasta samples showed a significantly (*p* < 0.05) higher protein content (8.22% and 10.7%, respectively) than the 100% cooked semolina fresh pasta (5.92%). In these samples, the loss of protein during the cooking process was about 27.7% for tagliatelle with 5% of GPP and only of 1.97% for those with 10% of GPP, underlining an improvement of the nutritional characteristics of the GPP-pasta samples after cooking with respect to 100% semolina fresh pasta. 

#### 3.2.2. Assessment of Polymeric Protein Distribution by SE-HPLC

**Figure 2:**
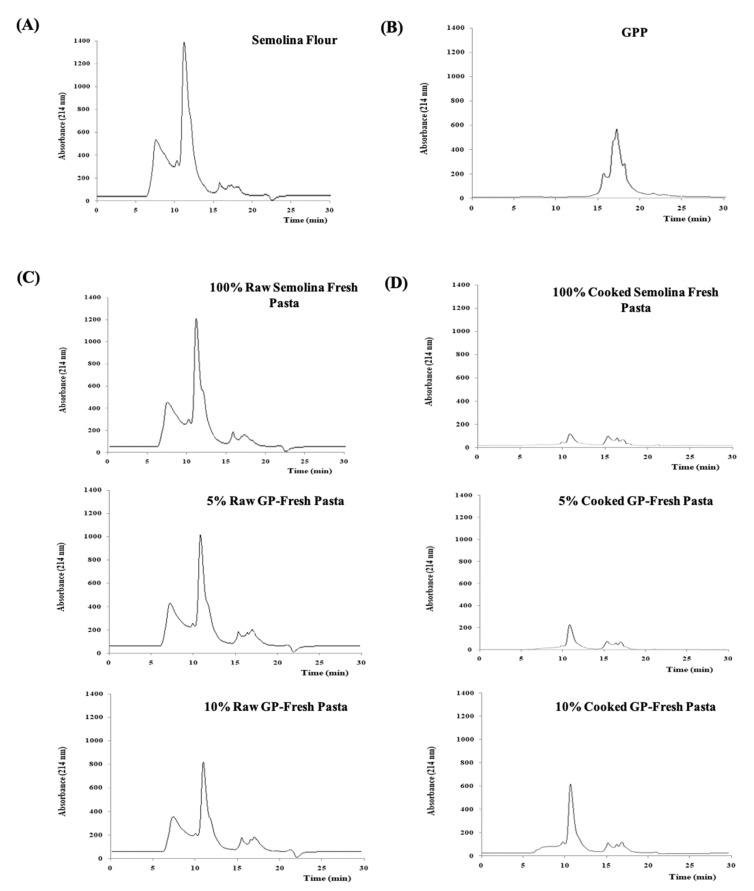
SE-HPLC profiles of SDS-extractable proteins of samples. (**A**): semolina flour; (**B**): freeze-dried GPP; (**C**): raw fresh pasta samples; (**D**): cooked fresh pasta samples.

Polymeric protein distribution of SDS-extractable proteins in semolina flour, freeze-dried grape pomace powder, and raw and cooked pasta samples are shown in Figure 2. Semolina flour (Figure 2A) showed a profile described by Lamacchia et al. [31,34], with a polymeric protein peak of glutenin with a high molecular weight (large polymeric proteins, LPP > 100.000 Da) after 7 min of elution, followed by a small peak of glutenins at a lower molecular weight (small polymeric proteins, included between 100.000 and 80.000 Da) between 8 and 10.5 min of elution, a large peak of monomeric gliadins (large monomeric proteins, LMP < 80.000 Da) (after 12 min of elution) and finally a small peak, including albumins and globulins, known as small monomeric proteins (SMP), comprised in the main peak of LMP. 

Freeze-dried GPP (Figure 2B) did not show SDS-extractable polymeric proteins in the considered range of molecular weights, because the elution of peaks after 15 min is attributable to the extraction solvent, according to la Gatta et al. [35].

The elution profile of raw pasta made with 100% semolina (Figure 2C), as well as those of raw GP-fresh pasta showed a profile very similar to semolina. On the contrary, significant differences in the number of peaks and peaks area were detectable in all the cooked pasta profiles (Figure 2D) (100% semolina, 5% and 10% GP-fresh pasta samples) with respect to the raw profiles. 

The SDS-extractable protein peak areas are shown in Table 3. As it can be inferred from the table, a significant decrease in all SDS-extractable proteins peaks area of cooked samples was detected, probably due to both the protein loss phenomenon, as shown in Table 2, and the polymerization reaction between different classes of gluten proteins during the cooking process, which led to the formation of SDS-unextractable polymeric proteins [30,31]. Among all the cooked pasta samples, the 10% GP one showed the highest total peaks area for SDS-extractable proteins, including all classes of gluten and non-gluten proteins, in line with Table 2, and thus confirming a different protein arrangement of this sample along the cooking process.

#### 3.2.3. Unextractable Polymeric Proteins (UPP) and S-S Bonds Formation Assessment

**Figure 3:**
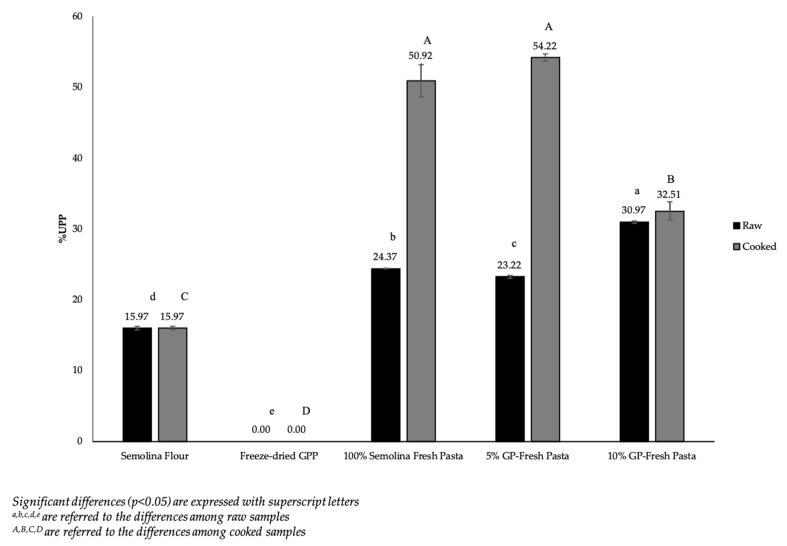
Unextractable polymeric proteins (%UPP) in raw and cooked pasta samples.

The strength of the gluten network was studied by evaluating both the proportion of “Unextractable Polymeric Proteins” (% UPP) and the amount of S–S groups formed during pasta making. The percentage of unextractable polymeric proteins (% UPP) in pasta samples was calculated from the peak areas of the SDS-extractable and SDS-unextractable proteins, obtained by proteins fractionation through SE-HPLC and are shown in Figure 3.

The formation of high-molecular-weight protein aggregates of cooked pasta samples was significantly higher (*p* < 0.05) than the raw samples, because of the formation of greater polymers between different classes of gluten and non-gluten proteins through covalent bonds during the cooking process [30,31]. In total, the 100% semolina pasta sample and 5% GP-fresh pasta sample showed the highest and similar values of UPP percentage for both raw and cooked samples (24.37% vs. 23.22% for raw samples and 50.92% and 54.22% for cooked samples, respectively). On the contrary, it was possible to note that the 10% GP-fresh pasta sample showed a significantly lower (*p* < 0.05) percentage of UPP, confirming a different protein arrangement of this sample, where the SDS-extractable fraction peak area was the highest, as shown in Table 3. This result was confirmed by the amount of S-S bonds (Table 4), which decreased significantly (*p* < 0.05) from 100% semolina pasta sample to that containing 10% of GPP in the cooked samples (36.19 to 3.44 nmol/mg of proteins, respectively). 

However, the decrease in S-S groups also corresponded to a significant decrease (*p* < 0.05) of SH groups, from 98.98 to 66.03 nmol/mg of proteins, comparing 100% cooked semolina pasta with 10% cooked GP-fresh pasta sample. These results suggested a possible interaction between the gluten proteins and polyphenols occurring in the GPP, also caused by non-disulfide covalent bonds. Moreover, high temperature increases polyphenols oxidation, which in turn, increases the reactivity and interaction with proteins. It is well established that reversible or irreversible covalent bonds can occur in protein–polyphenol conjugates between quinones or semiquinones, enhanced by oxidative and/or thermal conditions, and nucleophilic groups of proteins, such as the ε-amino group of -Lys and sulfhydryl (thiol) group of -Cys [36]. 

This interaction could explain the increase in the antioxidant activity in the cooked pasta samples, as shown in Table 1, since it is well known that protein-polyphenol interaction enhances the antioxidant activity of phenolic compounds. Furthermore, a protein modification through the interaction with other chemical components of food, such as phenolic compounds, has been identified as a promising approach for fabricating novel protein-based conjugates with enhanced antioxidant activity [36]. 

The interaction between gluten proteins and phenolic compounds could also explain the data of Table 2, in which the decrease in protein percentage after the cooking process in the 100% semolina fresh pasta, was significantly reduced (*p* < 0.05) in the fortified ones, proportionally with the increase in the grape pomace powder added. It could be supposed that the interaction and the formation of new complexes with polyphenols did not allow the proteins to be lost, as happened in 100% cooked semolina fresh pasta.

#### 3.2.4. Electrophoretic Profiles

**Figure 4:**
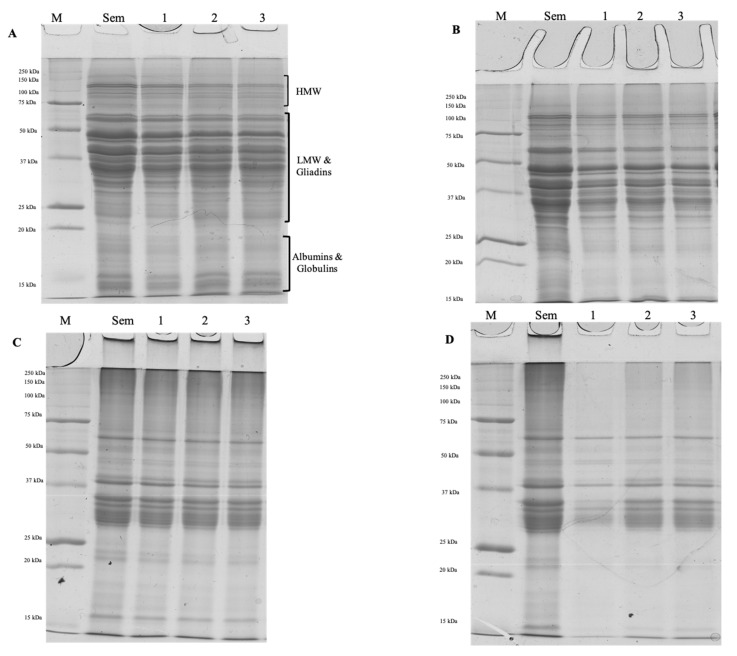
SDS-PAGE of fresh pasta samples. (**A**): Raw samples under reducing conditions; (**B**): Cooked samples under reducing conditions; (**C**): Raw samples under not reducing conditions; (**D**): Cooked samples under not reducing conditions. M: marker; SEM: semolina flour, 1: 100% Semolina fresh pasta; 2: 5% GP-fresh pasta; 3: 10% GP-fresh pasta.

For a better understanding of the protein decrease and the loss rate before and after the cooking procedure, the 100% semolina fresh pasta and GP-fresh pasta sample total proteins, for raw and cooked samples, were detected as CBB-stained bands on SDS-PAGE obtained under reducing and not reducing conditions (Figure 4).

Electrophoretic profiles of pasta samples under reducing conditions showed all the gluten proteins subunits (HMW > 90 KDa; LMW 60 < KDa > 14; gliadins 80 < KDa > 20 KDa) [33,36]. Raw and cooked pasta profiles under reducing conditions showed very similar patterns (Figure 4A,B), and no significant differences in the number and or band intensity were detectable.

Raw GP-fresh pasta profiles under not reducing conditions (Figure 4C) showed the presence of all semolina proteins (lane SEM) that looked very similar to semolina pasta (lane 1). Cooked pasta samples profiles under not reducing conditions (Figure 4D) showed, instead, a significant decrease in the intensity of the bands with respect to those detected for the raw samples (Figure 4C), especially in the case of 100% semolina fresh pasta, confirming once again the lower protein loss of the GP-fresh pasta samples after cooking (Table 2 and Figure 2). 

#### 3.2.5. Optimal Cooking Time

The cooking performance of the investigated fortified pasta samples in terms of optimal cooking time (OCT) are shown in Table 5. A significant difference among the OCT of the analyzed samples was observed, showing the highest value for the 10% GP- fresh pasta sample (4.55 min). Both the GP-fresh pasta samples showed a significant increase in the OCT with respect to the control sample (3.06 min). One possible explanation could be related to the capacity of the antioxidant compounds, occurring in GPP, to interact with gluten proteins, as suggested in Section 3.2.3, forming new complexes around the starch granules, encapsulating them during cooking and limiting the excessive swelling and diffusion of the amylose content [37], which increased the OCT.

### 3.3. Effect of the Addition of Freeze-Dried GPP on Fresh Semolina Tagliatelle Sensory Analysis

The sensory analysis allowed us to evaluate the acceptability of the new formulations of fresh pasta for consumers. Table 6 shows the mean values for each attribute of all tested raw and cooked pasta samples. No significant differences were observed among the samples for the tested parameters, even if the highest result for the overall acceptability of the raw samples was recorded in the 5% GP-fresh pasta (4.14 ± 0.09). This result was interesting because it highlighted the positive evaluation of the impact of the grape pomace addition in fresh pasta for the consumer. Considering that the control pasta (i.e., the 100% semolina fresh pasta) is commercially accepted, the data obtained showed that the new formulation would also be equally accepted. 

Additionally, it was evident that for cooked products there were differences among the samples (Table 6). For all the parameters, the newly formulated pasta samples showed a higher score than the control sample (i.e., 100% semolina fresh pasta), even though not significant. This could mean that cooking procedures made fortified pasta more acceptable, a crucial consideration when an innovative product has to be placed on the market. As a general feature, no significant differences were observed between pasta obtained with the addition of 5% or 10% of GPP.

**Figure 5:**
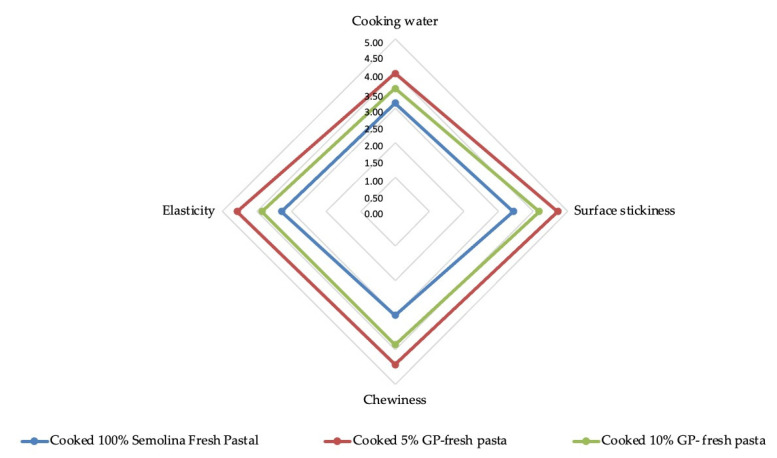
Spider plot with mean values for technical attributes evaluated for fresh pasta sample.

In order to evaluate which of the two new formulations was more appreciated, further parameters were evaluated. The spider plot (Figure 5) showed mean values for the most characteristic attributes evaluated for pasta samples. If Table 6 showed how the pasta products were accepted from a visual point of view, Figure 5 detailed how the consumer perceived the quality of the pasta after tasting it. It was important to highlight that the pasta product that achieved the highest score with a significant difference was 5% GP-fresh pasta. These results agreed with the fact that the addition of 10% of grape pomace in the formulation did not help to improve the formulation nor the protein organization, as the addition of 5% did.

## 4. Conclusions

The GP-fresh pasta samples showed, as expected, differences in the content of polyphenols, which increased proportionally with the percentage of GPP added, even if it was negatively affected by the cooking process. No differences were detected in the antioxidant activity of the raw GP-fresh pasta samples, which instead improved during the cooking process, probably due to interactions of polyphenols occurring in GPP with gluten proteins. Although, also in this case, the increase in the antioxidant activity was not proportional to the amount of GPP added. 

Gluten proteins–polyphenols interactions seemed to improve the chemical properties of newly formulated pasta samples, but also their nutritional and structural features, preventing protein loss during cooking, as happened in 100% semolina fresh pasta. Moreover, while the addition of 5% of GPP did not affect the organization of the gluten network in the raw and cooked GP-fresh pasta, a different rearrangement of the gluten structure was detectable with the addition of 10% of GGP. 

Nevertheless, Optimal Cooking time results confirmed the positive effect of the gluten network arrangement of both GP-fresh pasta samples. Finally, sensory analysis showed the preeminence of the 5% GP-fresh pasta sample for the desirable parameters for a cooked pasta, providing important information for the production of an innovative pasta product, in line with a sustainable production cycle. Further analyses are ongoing to better assess the chemical and microbiological characteristics of the newly formulated pasta samples.

## 5. Patents

Patent no. 001426984 was used in this work.

## Figures and Tables

**Table 1:** Total polyphenolic content (TPC) with Folin–Ciocalteu and Antioxidant activity with DPPH method of grape pomace powders (GPP) (a) and fresh pasta samples (b).

(**a**)		
***Samples***	**TPC** **(mg of Gallic Acid/100 g)**	**Antioxidant Activity** **(μmol TEAC/100 g)**
Raw GPP	3191 ± 6 ^c^	1417 ± 67 ^a^
Dried GPP	7380 ± 267 ^b^	1303 ± 86 ^a^
Freeze-dried GPP	18199 ± 240 ^a^	1523 ± 82 ^a^
(**b**)		
**Samples**	**TPC** **(mg of Gallic Acid/100 g)**	**Antioxidant Activity** **(μmol TEAC/100 g)**
Semolina Flour	143 ± 5 ^c^	0 ^c^
100% Raw Semolina Fresh Pasta	53 ± 3.8 ^d^	0 ^c^
100% Cooked Semolina Fresh Pasta	61.1 ± 2.5 ^d^	0 ^c^
5% Raw GP-Fresh Pasta	219 ± 10 ^b^	1933 ± 60 ^b^
5% Cooked GP-Fresh Pasta	150 ± 3 ^c^	2199 ± 28 ^a^
10% Raw GP-Fresh Pasta	305 ± 2 ^a^	1913 ± 96 ^b^
10% Cooked GP-Fresh Pasta	202 ± 4 ^b^	2160 ± 22 ^a^

^a, b, c, d^ (*p* < 0.05) Significant differences are expressed with superscript letters within a column for each parameter.

**Table 2:** Content of total protein expressed as percentage of protein (g/100 g).

Sample	%Protein
Semolina flour	12.0 ± 0.01 ^a^
Freeze-dried GPP	10.5 ± 0.01 ^f^
100% Raw Semolina Fresh Pasta	11.6 ± 0.03 ^b^
100% Cooked Semolina Fresh Pasta	5.92 ± 0.02 ^h^
5% Raw GP-Fresh Pasta	11.3 ± 0.04 ^c^
5% Cooked GP-Fresh Pasta	8.22 ± 0.02 ^g^
10% Raw GP-Fresh Pasta	10.9 ± 0.04 ^d^
10% Cooked GP-Fresh Pasta	10.7 ± 0.06 ^e^

^a, b, c, d, e, f, g, h^ (*p* < 0.05) Significant differences are expressed with superscript letters within a column for each parameter.

**Table 3:** Peaks area of SDS-extractable proteins for semolina flour, grape pomace powder, and raw and cooked pasta samples.

		Semolina Flour	Grape Pomace Powder	100% Raw Semolina Fresh Pasta	100% Cooked Semolina Fresh Pasta	5% Raw GP-Fresh Pasta	5% Cooked GP-Fresh Pasta	10% Raw GP-Fresh Pasta	10% Cooked GP-Fresh Pasta
**Peak 1**	mAu⋅s %	42652.50 ± 533.87 *21.43*	/	35610.90 ± 53.74 *23.90*	161.55 ± 48.44 *2.18*	25712.60 ± 1800.01 *23.07*	170 ± 5.59 *1.06*	23641.60 ± 268.28 *25.44*	942.90 ± 190.78 *2.13*
**Peak 2**	mAu⋅s %	39433.40 ± 19.65 *19.82*	/	*28121.95* ± 1967.81 *18.88*	1667.25 ± 301.16 *22.45*	23552.30 ± 1643.32 *21.13*	3049.20 ± 36.91 *18.99*	19221.95 ± 330.57 *20.69*	10199.65 ± 150.26 *22.99*
**Peak 3**	mAu⋅s %	118564.80 ± 754.48 *39.01*	/	*55275.15* ± 1665.45 *37.10*	5596.70 ± 11.17 *75.37*	44414.05 ± 422.35 *39.86*	12837.25 ± 141.92 *79.95*	36322.05 ± 977.15 *39.09*	33224.70 ± 467.68 *74.89*
**Peak 4**	mAu⋅s	39275.80 ± 3487.31 *19.74*	/	*29973.65* ± 995.82 *20.12*	/	17759.60 ± 2073.24 *15.94*	/	13736.05 ± 190.85 *14.78*	/

**Table 4:** Content in free thiol (SH) and disulfide groups (SS) in pasta samples. Mean ± standard error for three replicates. Values are nanomoles per milligram of protein.

Samples	Free Thiol (nmol/mg Prot)	Disulfide Groups (nmol/mg Prot)
Semolina Flour	70.56 ± 0.68 ^b,c^	33.65 ± 2.84 ^a^
100% Raw Semolina Fresh Pasta	63.98 ± 2.80 ^c^	17.98 ± 3.50 ^b,c^
100% Cooked Semolina Fresh Pasta	98.98 ± 7.68 ^a^	36.19 ± 4.12 ^a^
5% Raw GP-Fresh Pasta	69.03 ± 2.14 ^b,c^	21.26 ± 3.42 ^b^
5% Cooked GP-Fresh Pasta	78.55 ± 2.37 ^b^	8.11 ± 3.16 ^d,e^
10% Raw GP-Fresh Pasta	68.30 ± 1.49 ^b,c^	12.49 ± 1.34 ^c,d^
10% Cooked GP-Fresh Pasta	66.03 ± 2.89 ^b,c^	3.44 ± 3.34 ^e^

^a, b, c, d, e^ Significant differences (*p* < 0.05) are expressed with superscript letters within a column for each parameter.

**Table 5:** Optimal cooking time (OCT) of fresh pasta samples.

Samples	OCT (min)
100% Semolina Fresh Pasta	3.06 ± 0.13 ^c^
5% GP-Fresh Pasta	4.03 ± 0.06 ^b^
10% GP-Fresh Pasta	4.55 ± 0.10 ^a^

^a, b, c^ (*p* < 0.05).

**Table 6:** Sensory analysis of raw and cooked fresh pasta samples.

Raw Fresh Pasta			
	100% Semolina Fresh Pasta	5% Grape Pomace Fresh Pasta	10% Grape Pomace Fresh Pasta
Odor	3.71 ±0.12	3.71 ± 0.04	3.43 ± 0.28
Color	3.57 ± 0.76	3.86 ± 0.89	3.71 ± 1.06
Superficial aspect	3.86 ± 0.14	4.00 ± 0.16	3.86 ± 0.19
General presentation	3.71 ± 0.12	3.71 ± 0.04	4.00 ± 0.09
Overall acceptability	3.86 ± 0.09	4.14 ± 0.09	4.00 ± 0.04
**Cooked Fresh Pasta**			
	**100% Semolina Fresh Pasta**	**5% Grape Pomace Fresh Pasta**	**10% Grape Pomace Fresh Pasta**
Odor	3.86 ± 0.1	4.00 ± 0.03	4.46 ± 0.08
Color	3.71 ± 0.04	4.14 ± 0.04	4.61 ± 0.07
Superficial aspect	3.71 ± 0.06	4.43 ± 0.02	4.61 ± 0.07
General presentation	4.00 ± 0.06	4.14 ± 0.04	4.75 ± 0.05
Overall acceptability	3.71 ± 0.04	4.57 ± 0.02	4.79 ± 0.08

## Data Availability

Data are contained within the article and Supplementary Materials.

## Supplementary Materials

The following supporting information can be downloaded at: https://www.mdpi.com/article/10.3390/foods12142699/s1, Figure S1. HPLC-DAD polyphenolic profiles of grape pomace powders (GPP) at 280, 313 nm. Panel A, B: Raw GPP; Panel B, C: Dried GPP; Panel E, F: Freeze-dried GPP.
