# Supplementary material for: Effect of the Addition of Freeze-Dried Grape Pomace on Fresh Tagliatelle Gluten Network and Relationship to Sensory and Chemical Quality
Source: Foods. 2023 Jul 13;12(14):2699. doi: 10.3390/foods12142699 (PMC10378643; doi:10.3390/foods12142699)
Supplement: Supplementary file 1 [file foods-12-02699-s001.zip › Figure S1.pptx]

## Slide 1
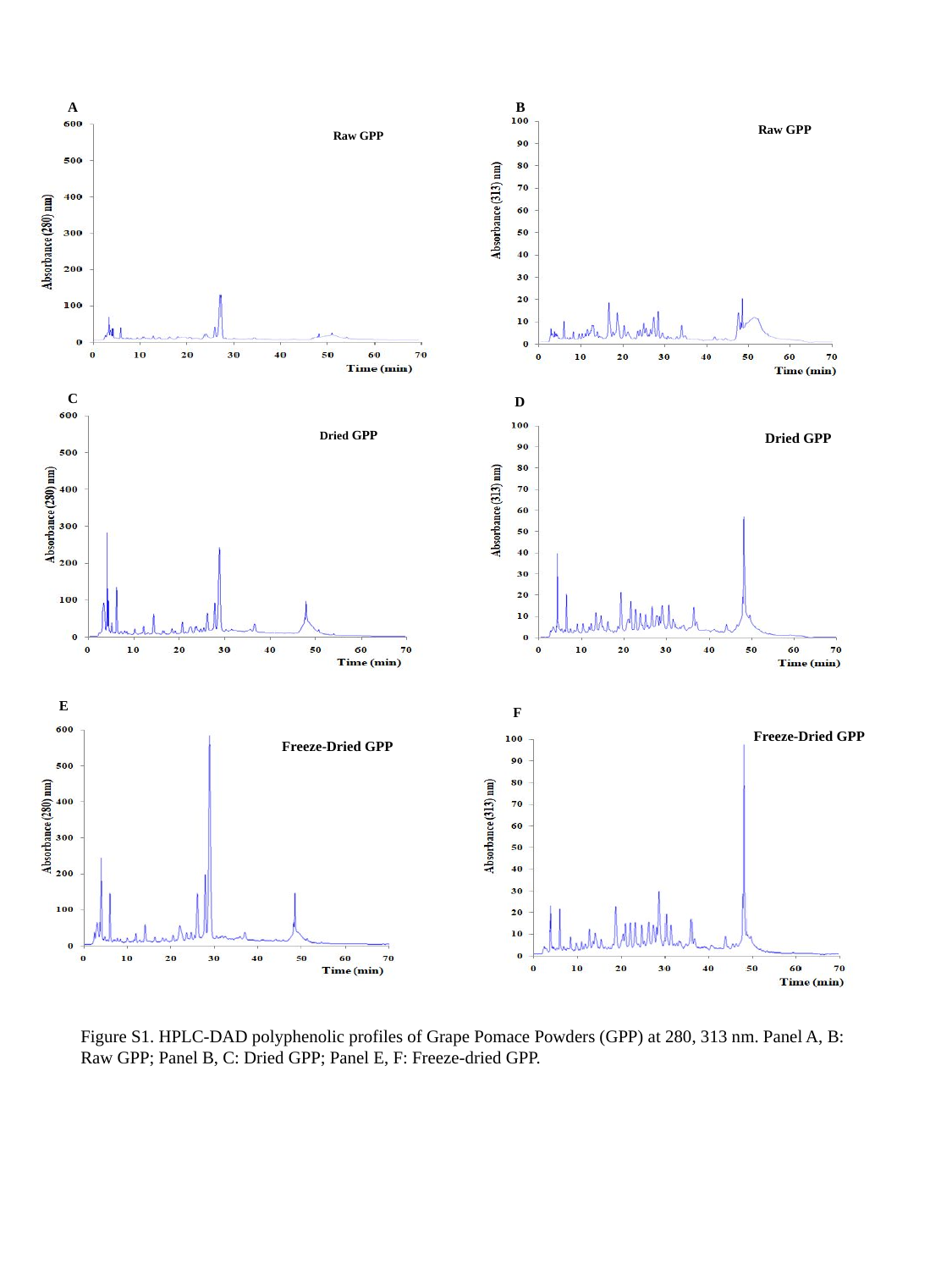

A
B
Raw GPP
Raw GPP
C
D
Dried GPP
Dried GPP
E
F
Freeze-Dried GPP
Freeze-Dried GPP
Figure S1. HPLC-DAD polyphenolic profiles of Grape Pomace Powders (GPP) at 280, 313 nm. Panel A, B: Raw GPP; Panel B, C: Dried GPP; Panel E, F: Freeze-dried GPP.
